# Two Unusual Presentations of Presacral Schwannoma; A Case Series

**DOI:** 10.1016/j.ijscr.2019.07.042

**Published:** 2019-07-22

**Authors:** Dana Kalagi, Mohammed Bakir, Mohammad Alfarra, Alaa Aborayya, Ihab Anwar

**Affiliations:** a Alfaisal University, College of Medicine, Riyadh, Saudi Arabia; b Department of Surgery, Section of General and Oncology Surgery, King Faisal Specialist Hospital & Research Centre, Riyadh, Saudi Arabia

**Keywords:** Presacral schwannoma, Retroperitoneal, Laparoscopic, Case series

## Abstract

**Introduction:**

Schwannomas are benign tumors that arise from the Schwann cells of nerve fibers. They commonly occur in the head and neck, mediastinum and extremities. However, they are extremely rare to be found in the pelvis.

**Case presentation:**

We report two cases of pelvic presacral schwannomas found in our hospital. The first case is a 50 years old lady who was seen in the outpatient clinic complaining of pelvic heaviness. Investigations were done including Computed Tomography (CT) and Magnetic Resonance Imaging (MRI) which showed a solid mass in the presacral area. Biopsy was taken, and results came back as schwannoma. The patient underwent exploratory laparotomy and total tumor resection. The second case is a 19 years old gentleman who was referred from a local hospital to our hospital for further evaluation of an asymptomatic left retroperitoneal pelvic mass. The mass was found incidentally after a Road Traffic Accident. The patient underwent exploratory laparotomy and total tumor resection. In the two cases, specimen histopathology report came back positive for schwannoma and patients were doing fine in the post-operative period with no signs of recurrence till this date.

**Conclusion:**

Due to the few cases in the literature, the diagnosis of presacral schwannoma is challenging. Some patients presented with vague symptoms like pelvic heaviness as seen with the patient in the first case, and some presented completely asymptomatic as seen with the patient in the second case. In symptomatic and asymptomatic cases, surgical excision is the mainstay of treatment of these tumors.

## Highlights

• Schwannomas are benign tumors that commonly occur in the head and neck, mediastinum and extremities.
• Pelvic schwannomas are very rare.
• Patients with pelvic schwannomas have different clinical presentations.
• We reported 2 cases of schwannomas found at the presacral area.
• The mass was found incidentally in one of the cases and the patient was completely asymptomatic.

## 1 Introduction

Schwannomas are benign tumors that arise from the Schwann cells of nerve fibers. They commonly occur in the head and neck, mediastinum and extremities. They are extremely rare to be found in the pelvis accounting for 1–3% of all schwannomas and less than 0.5% of reported cases, unless they are combined with von Recklinghousen disease (type 1 neurofibromatosis) [1,2]. These tumors are nonaggressive, slow growing, solitary neoplasms with an extremely low possibility of malignant transformation or recurrence after excision [2].

Schwannomas can rarely affect the gastrointestinal tract and visceral organs. They are in general asymptomatic and may grow to a considerable size before becoming symptomatic if they are located in deep-seated structures like the retroperitoneal area. They become symptomatic by local compression of the surrounding viscera or by nerve root compression leading to the nonspecific presenting signs and symptoms such as backache, abdominal or pelvic heaviness or distension [3]. These vague symptoms can mimic a number of pelvic lesions which make pelvic schwannomas easily misdiagnosed, making radiographic imaging essential for the preoperative evaluation to obtain information about tumor size, location and relationships with neighboring tissues to determine the optimal surgical approach and the extent of surgical resection [4,5]. The main treatment is considered to be complete excision of the tumor either laparoscopically or with open abdominal surgery [2].

In our case report, we are presenting two rare presentations of Schwannoma in the presacral area that was treated by surgical excision. The work has been reported in line with the PROCESS criteria [6]. This series project was registered with research registry (4945) and obtained ORA approval from our institute n. 2,190,022.

## 2 Case presentation

### 2.1 Case 1

**Fig. 1:**
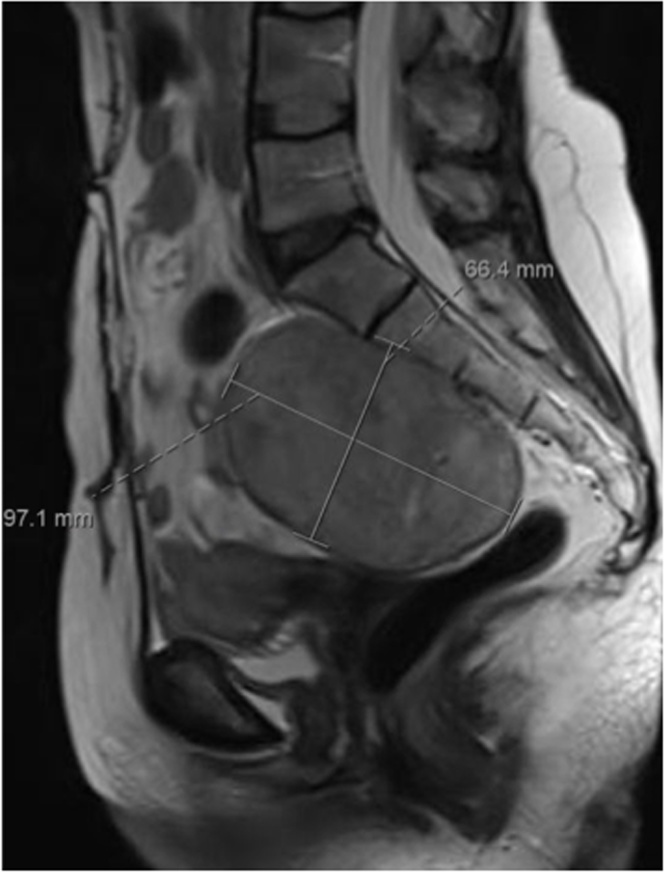
MRI of pelvis with contrast showing a large encapsulated mass located at the presacral region from S1 to S3 measures 7.5 × 6.6 × 9.7 cm in transverse, AP, and long axis respectively.

**Fig. 2:**
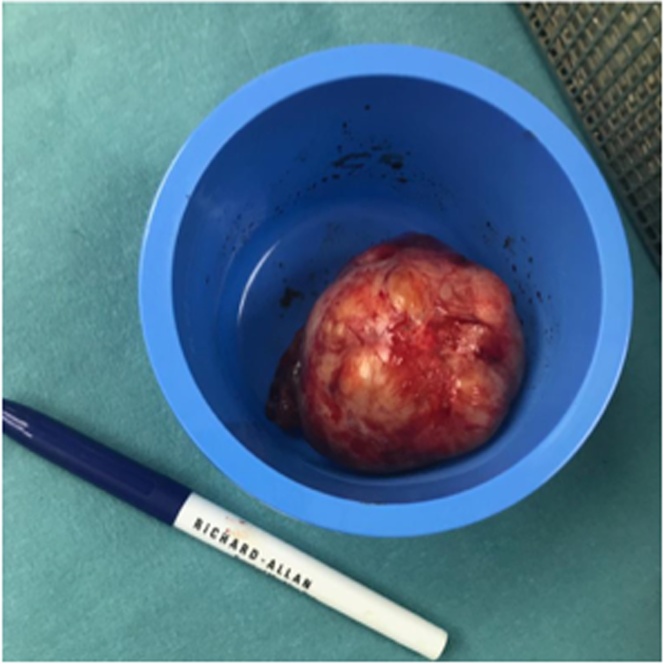
Macroscopic image showing the large mass after resection.

**Fig. 3:**
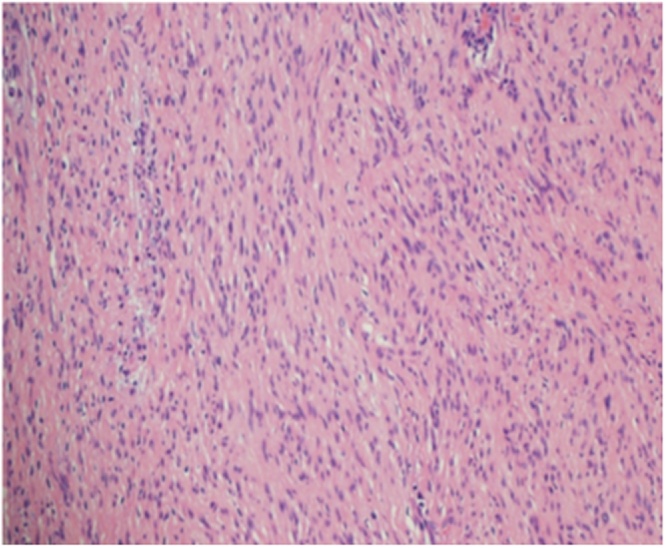
Histopathology slide showing spindle cell lesion consistent with schwannoma.

A 50 year old women was seen in the outpatient clinic complaining of heaviness in the pelvic area that started one year ago and progressing with time. The patient had no genitourinary symptoms, no constitutional symptoms, and no history of trauma. Physical examination was normal with no palpable masses or abnormalities. Investigations were done including CT scan and MRI which showed a solid mass in the presacral area (Fig. 1). CT guided true cut biopsy was taken and came back positive for schwannoma. The patient was booked for surgery as a case of a large presacral mass for exploratory laparotomy and total tumor resection. The tumor was excised (Fig. 2), and histopathology report confirmed the diagnosis of Schwannoma (Fig. 3).

### 2.2 Case 2

**Fig. 4:**
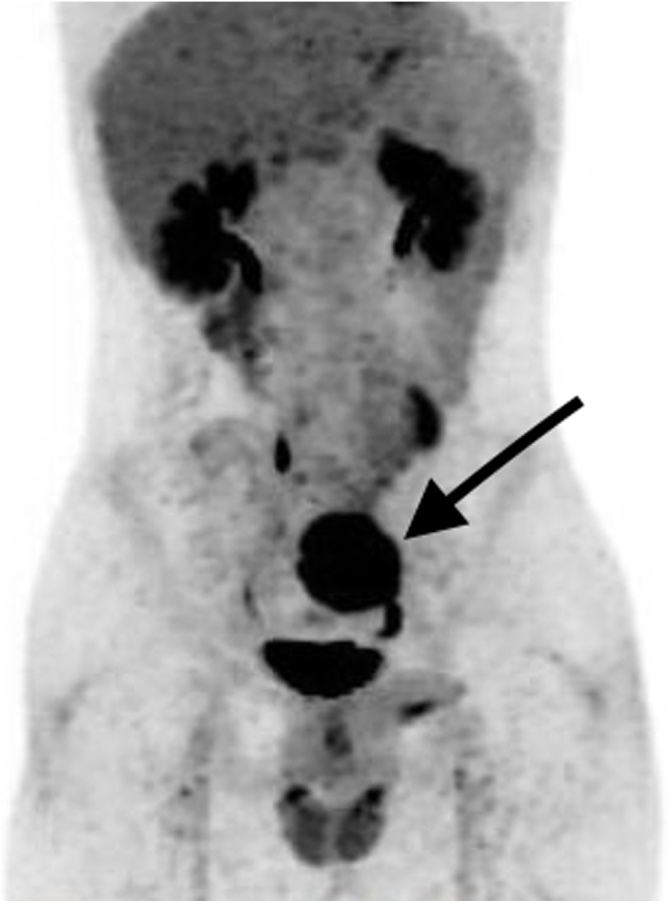
PET scan showing moderate FDG avidity within well circumscribed rounded mass in the left pelvis measures 6.2 × 5.7 cm axially.

**Fig. 5:**
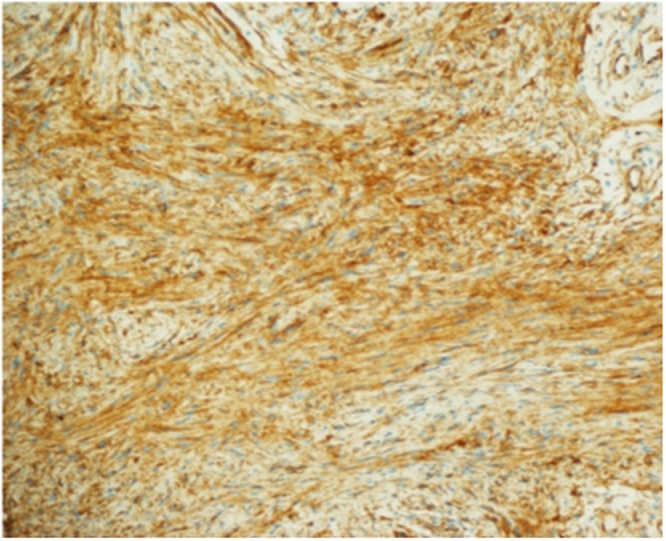
Histopathology slide showing spindle cell lesion with diffuse s100 staining consistent with schwannoma.

A 19 Year old man medically free, referred from a local hospital to our hospital for further evaluation of an asymptomatic left retroperitoneal pelvic mass. The mass was found incidentally after a Road Traffic Accident, otherwise the patient was completely asymptomatic. The CT scan done outside the hospital was reviewed and Positron Emission Tomography (PET) scan was done in our hospital for a better evaluation of the mass to determine whether this mass is malignant or benign and to establish baseline staging. The PET scan showed a mass in the left pelvic area demonstrating a moderate uptake with possible malignant process (Fig. 4). The patient couldn’t go for a CT guided true cut biopsy due to the difficult position of the mass as explained by the interventional radiologist, as well as the patient’s body built and narrow pelvis. The patient then was scheduled for exploratory laparotomy and total tumor resection. The tumor was excised, and histopathology report came positive for the diagnosis of Schwannoma (Fig. 5).

## 3 Discussion

Pelvic schwannomas are soft tissue tumors that originate from a sacral nerve or the hypogastric plexus and account for only a small fraction of schwannoma cases as reported [7]. Due to the fact that pelvic schwannomas are slow growing in nature, patients remain asymptomatic and tumors are usually found incidentally during medical investigations for unrelated symptoms. They only become symptomatic when the tumor has grown in size to cause the mass effect. This mass effect can lead to pain in the pelvic area and lower back and a sense of heaviness accompanied with urinary and digestive symptoms caused by bladder and bowel compression due to the limited small space [2]. In one of the case series found in the literature, only 1 individual, whose schwannoma was causing sacral destruction, presented with pain, whereas in 5 cases the masses were discovered incidentally during unrelated investigations or procedures [8].

Preoperative diagnosis of schwannoma is challenging due to the lack of distinguishing features on imaging studies between benign and malignant schwannomas as well as schwannomas and other soft tissue tumors such as fibrosarcoma and liposarcomas [8]. Although it’s not always accurate, ultrasound- guided biopsy of large pelvic tumors is suggested for the preoperative diagnosis, as the definitive treatment is mainly based on the histopathological findings preoperatively [7]. In the first case we presented, the preoperative diagnosis was made by the true cut biopsy, which was helpful to determine if the mass was benign or malignant before proceeding to surgery in order to know the extent of dissection needed. In the second case we presented, a true cut biopsy was difficult to obtain.

For the treatment of pelvic presacral schwannomas, as most of the retroperitoneal tumors are in an anatomically complex and located in an inaccessible site with surrounding vital structures, it may be hard to obtain a complete resection with sufficient clear margins through laparoscopic approach as advised in the literature [7]. The laparoscopic approach was not a good option in our cases. In the first case, the mass was adherent to the sacral bone which made us go for the open approach. In the second case, since preoperative diagnosis couldn’t be made, and due to his very narrow pelvis the open approach was beneficial for the better evaluation and visualization of the mass without the risk of injuring the adjacent vessels and structures. Intraoperatively, the mass was not adherent to the sacral bone, covered by peritoneum, and not invading the surrounding tissue giving the impression of a benign tumor, in contrast to the PET scan findings preoperatively.

## 4 Conclusion

Pelvic masses are common, but due to the small number of reported cases of pelvic schwannomas and the rarity of these tumors, presacral schwannoma was not on top of the differential diagnosis in the two cases we are reporting. Since signs and symptoms aren’t specific and due to the lack of pathognomonic findings on radiological imaging, biopsy is considered the best diagnostic approach to direct the surgeon for further management.

## Funding

The authors declare no source of funding.

## Ethical approval

Approval has been given by ORA at our institute.

## Consent

Written informed consent was obtained from the patient for publication of this case report and accompanying images. A copy of the written consent is available for review by the Editor-in-Chief of this journal on request.

## Author contribution

Dana Kalagi: Literature review and writing the manuscript.

Mohammed Bakir: Literature review and writing the manuscript.

Mohammad Alfarra: writing and reviewing the manuscript.

Alaa Aborayya: writing and reviewing the manuscript.

Ihab Anwar: Performing surgeon and reviewing the manuscript.

## Registration of research studies

This series project was registered with research registry (4945) and obtained ORA approval from our institute n. 2,190,022.

## Guarantor

Dana Kalagi.

## Provenance and peer review

Not commissioned, externally peer-reviewed.

## Declaration of Competing Interest

The authors declare no conflict of interest.
